# Predicting mucosal healing in Crohn’s disease: development of a deep-learning model based on intestinal ultrasound images

**DOI:** 10.1186/s13244-025-02014-5

**Published:** 2025-06-16

**Authors:** Li Ma, Yuepeng Chen, Xiangling Fu, Jing Qin, Yanwen Luo, Yuanjing Gao, Wenbo Li, Mengsu Xiao, Zheng Cao, Jialin Shi, Qingli Zhu, Chenyi Guo, Ji Wu

**Affiliations:** 1 Department of Ultrasound, Peking Union Medical College Hospital, Chinese Academy of Medical Sciences, Beijing, China; 2 School of Computer Science (National Pilot Software Engineering School), Beijing University of Posts and Telecommunications, Beijing, China; 3 BirenTech Research, Shanghai, China; 4 Department of Electronic Engineering, Tsinghua University, Beijing, China

**Keywords:** Crohn’s disease, Outcome prediction, Deep learning, Intestinal ultrasound.

## Abstract

**Objective:**

Predicting treatment response in Crohn’s disease (CD) is essential for making an optimal therapeutic regimen, but relevant models are lacking. This study aimed to develop a deep learning model based on baseline intestinal ultrasound (IUS) images and clinical information to predict mucosal healing.

**Methods:**

Consecutive CD patients who underwent pretreatment IUS were retrospectively recruited at a tertiary hospital. A total of 1548 IUS images of longitudinal diseased bowel segments were collected and divided into a training cohort and a test cohort. A convolutional neural network model was developed to predict mucosal healing after one year of standardized treatment. The model’s efficacy was validated using the five-fold internal cross-validation and further tested in the test cohort.

**Results:**

A total of 190 patients (68.9% men, mean age 32.3 ± 14.1 years) were enrolled, consisting of 1038 IUS images of mucosal healing and 510 images of no mucosal healing. The mean area under the curve in the test cohort was 0.73 (95% CI: 0.68–0.78), with the mean sensitivity of 68.1% (95% CI: 60.5–77.4%), specificity of 69.5% (95% CI: 60.1–77.2%), positive prediction value of 80.0% (95% CI: 74.5–84.9%), negative prediction value of 54.8% (95% CI: 48.0–63.7%). Heat maps showing the deep-learning decision-making process revealed that information from the bowel wall, serous surface, and surrounding mesentery was mainly considered by the model.

**Conclusions:**

We developed a deep learning model based on IUS images to predict mucosal healing in CD with notable accuracy. Further validation and improvement of this model with more multi-center, real-world data are needed.

**Critical relevance statement:**

Predicting treatment response in CD is essential to making an optimal therapeutic regimen. In this study, a deep-learning model using pretreatment ultrasound images and clinical information was generated to predict mucosal healing with an AUC of 0.73.

**Key Points:**

- Response to medication treatment is highly variable among patients with CD.
- High-resolution IUS images of the intestinal wall may hide significant characteristics for treatment response.
- A deep-learning model capable of predicting treatment response was generated using pretreatment IUS images.

**Graphical Abstract:**

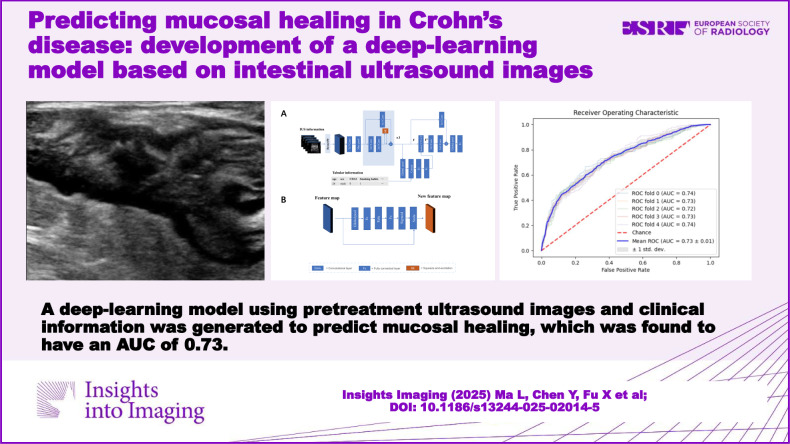

## Introduction

Crohn’s disease (CD) is a chronic inflammatory bowel disease. Rapid development has occurred in CD medications over the past decade, especially multiple novel biologic therapies, which have significantly improved the prognosis of CD patients [1]. However, the response to medication is highly variable among patients, and often appears random [2]. Considering the potential side effects and the high cost of these drugs, there is an increasing need to develop accurate models that can reliably allow early prediction of mucosal healing, a landmark of treatment success [3]. The ability to predict treatment response would enable personalized and precise patient care, ultimately improving the long-term outcomes for CD patients [2].

Various demographic, biochemical, and genomic factors, including onset age, smoking, fecal calprotectin levels, apoptotic gene polymorphisms, circulating cytokines, miRNAs, have been tried to predict mucosal healing via traditional statistical methods [4–6]. Despite numerous studies, no prediction models have been successfully developed for clinical use. New potential predictive factors, coupled with novel analytical methods, such as serological markers, genomics, and artificial intelligence, can be the new breakthrough and are waiting to be explored.

Intestinal ultrasound (IUS) is an indispensable imaging modality for evaluating intestinal conditions in CD. The European Crohn’s and Colitis Organisation and the European Society of Gastrointestinal and Abdominal Radiology (ECCO-ESGAR) guidelines recommend IUS as the first-line imaging test for assessing and monitoring CD [7]. Recent studies have identified an association between pretreatment IUS characteristics and the treatment response, indicating the potential predictive value of IUS [4, 8, 9]. One advantage of IUS is its ability to provide a detailed view of the intestinal wall, facilitating the examination of wall morphology, vascularity, and motility, thereby enabling the extraction of more detailed information [10].

Recently, deep learning methods have shown promising roles in diagnosing or predicting treatment responsiveness in various healthcare conditions, including CD [11]. Unlike human eyes, which can only determine rough morphological characteristics such as bowel wall stratification and semi-quantitative vascularity grades, deep learning techniques can extract detailed information from diseased intestinal walls, potentially allowing researchers to assess various treatment responses. However, studies using IUS images to predict mucosal healing with deep learning methods are lacking.

To develop a practical method that may facilitate the improvement and individualization of CD treatment, this study aimed to develop a deep learning model capable of predicting mucosal healing based on baseline IUS images, along with the clinical, laboratory, and endoscopic data.

## Materials and methods

### Patients and study design

This retrospective cohort study was conducted at a single tertiary hospital and approved by the Institutional Review Board (IRB) of this hospital. Written informed consent was waived by the IRB. Consecutive patients with CD who were hospitalized between September 2015 and February 2022 were eligible for study inclusion. Inclusion criteria were as follows: (1) confirmed CD diagnosis based on ECCO guidelines [12, 13]; (2) received transabdominal IUS and endoscopy examinations at baseline; (3) underwent a standardized therapeutic regimen conforming to ECCO-ESGAR guidelines, and underwent routine evaluations throughout a follow-up period of ≥ 12 months. Exclusion criteria were as follows: (1) incomplete evaluation of intestinal lesions by IUS or endoscopy; (2) underwent gastrointestinal surgeries or interventions during the follow-up period; (3) pregnancy, severe systemic comorbidities, or bowel comorbidities including malignant intestinal tumors, Behcet’s disease, or intestinal tuberculosis.

Demographic information, clinical course, Montreal classification, smoking status, medication history, surgical history, C-reactive protein (CRP), albumin (Alb), CDAI, and Simplified Endoscopic Score for Crohn’s Diseas (SES-CD) were collected at baseline. Patients then undertook anti-inflammatory therapy and were regularly followed up for at least one year, with reassessments based on symptoms, biochemical markers, endoscopy, and imaging studies. Colonoscopy was performed at least 12 months (52 weeks) after treatment initiation. For patients with small intestine involvement, capsule or balloon-assisted small intestine endoscopy was additionally performed. In our cohort, 67 patients had small bowel lesions, with 500 IUS images involving the small intestine.

### IUS examination and image selection

One of the three radiologists with > 10 years of intestinal imaging experience performed IUS studies using a Philips iU22 (Philips Healthcare) or SuperSonic Aixplorer (SuperSonic Imaging) machine with convex (C5-2) and linear (L9-3) transducers, following the guidelines of the European Federation of Societies for Ultrasound in Medicine and Biology (EFSUMB) [14]. Patients were instructed to fast for at least 8 h prior to IUS examination. The colon was scanned along its route, and longitudinal gray-scale IUS images were captured consecutively, each representing a 5-cm bowel segment. For the small intestine, a mowing-lawn scanning method was performed, and only images of diseased lesions were saved. In total, 20–30 IUS images were saved for each examination.

**Fig. 1:**
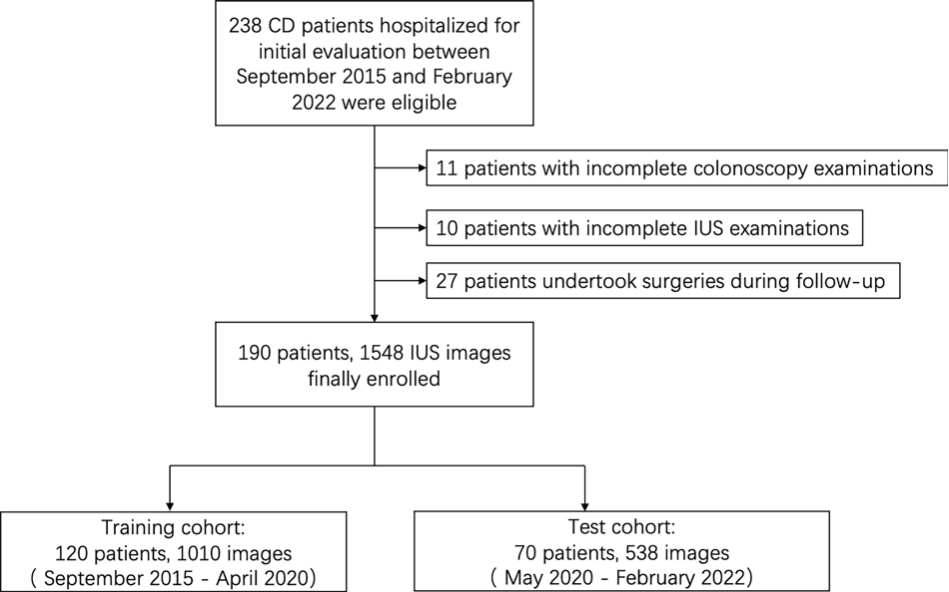
A flow diagram describing patient enrollment. CD, Crohn’s disease; IUS, intestinal ultrasound

Bowel wall thickness (BWT) was consecutively measured at the terminal ileum, ileocecal area, ascending colon, transverse colon, descending colon, and sigmoid colon with linear transducers. BWT measurements of each bowel section were performed at least twice, and the mean value was recorded. A BWT > 3 mm was considered indicative of disease. All longitudinal gray-scale images of the diseased bowel segments were selected for machine learning [15]. Image selection was meticulously performed by one radiologist with more than 10 years of experience in abdominal imaging to optimize data quality according to clear visualization of the bowel wall along the longitudinal axis and minimal artifacts, including motion blur or acoustic shadowing. Due to the operator-dependent nature of ultrasound acquisition, there was a variation in the number of non-qualified images across examinations. Nevertheless, a minimum of one eligible image per affected site was ensured for further analysis. Images of extraintestinal complications, such as fistulas and abscesses, were also included. Simultaneous CT was usually performed to cross-check the extraintestinal findings. IUS images were exported from the ultrasound machine and saved in JPEG. format with the highest resolution. For each patient, 1–25 IUS static images with diseased bowel segments were chosen, resulting in a total of 1548 IUS images. All selected diseased bowel segments on IUS images were confirmed with the presence of ulcerations or erosions under endoscopy. Based on the examination dates, the 1548 images were divided into a training cohort (September 2015–April 2020) and a test cohort (May 2020–Februry 2022) (Fig. 1).

For comparison, we also calculated the International Bowel Ultrasound Segmental Activity Score (IBUS-SAS), an objective scoring system incorporating IUS parameters such as BWT, stratification, inflammatory fat, and vascularization as previously reported [16].

### Ground truth assessment

We defined mucosal healing/no mucosal healing at the follow-up assessment using endoscopy as the ground truth for deep learning. Mucosal healing was determined as the absence of active inflammation upon endoscopy (SES-CD of 0). The location correlation of baseline IUS images and follow-up endoscopy was performed by one gastroenterologist and one radiologist together, who reviewed IUS images and picked the corresponding endoscopic images/videos. Another gastroenterologist who was blinded to the clinical, laboratory, and IUS data independently evaluated the picked endoscopic images/videos for mucosal healing or not. Baseline IUS images were classified into “mucosal healing” or “no mucosal healing” based on the correlated endoscopic results.

On the patient level, if no active inflammation was observed in any bowel segments (SES-CD of 0), the patient was considered to have achieved mucosal healing [17]. This was evaluated by one gastroenterologist based on the whole follow-up endoscopic images/videos of one patient, who was blinded to the clinical, laboratory, and IUS data.

### Data preprocessing for deep learning

We uniformly resized the collected IUS images to 512 × 1024 pixels and standardized the pixel values within a range of −1 to 1. Relevant information in the images, including the intestinal wall, fistulas, and abscesses, was outlined using the software Colabeler (Kuaiyi Technology) by one of the four radiologists who were blinded to the follow-up results to prevent the introduction of confounding information to the model. To expand the dataset, images were flipped horizontally, vertically, and randomly.

To improve the performance of the prediction model, tabular clinical and laboratory information, including gender, age at enrollment, disease duration, disease location, CDAI, hypersensitive C-reactive protein (hsCRP), smoking, previous bowel surgery, and medication, was also collected alongside IUS images (Table 1). For tabular information, different encoding formats were used to represent model features according to data type. Discrete data were represented using one-hot encoding, while continuous data were represented using numerical values. Finally, features were normalized and incorporated into the deep learning model.

**Table 1:** Table characteristics of patients and IUS images

	Patients (*N* = 190)	Images (*N* = 1548)	Training cohort (1010 images from 120 patients)	Test cohort (538 images from 70 patients)	*p*
Sex (male:female)	131:59	1055:493	727:283	328:210	0.20
Age at enrollment, years (mean ± SD)	32.3 ± 14.1	32.5 ± 14.1	31.8 ± 14.3	33.8 ± 13.6	0.28
Disease duration, months (mean ± SD)	73.4 ± 58.1	68.1 ± 54.5	68.4 ± 49.4	67.5 ± 63.0	0.12
Age at diagnosis, *n* (%)					0.56
A1 (≤ 16 y)	35 (18.4)	252 (16.3)	158 (15.6)	98 (18.2)	
A2 (17–40 y)	118 (62.1)	926 (59.8)	618 (53.7)	329 (61.2)	
A3 (> 40 y)	37 (19.5)	370 (23.9)	234 (23.2)	185 (20.6)	
Location, *n* (%)					0.81
L1 (terminal ileum)	42 (22.1)	337 (21.8)	222 (22.0)	102 (19.4)	
L2 (colon)	43 (22.6)	299 (25.8)	179 (17.7)	152 (28.2)	
L3 (ileocolon)	105 (55.3)	912 (58.9)	609 (60.3)	284 (52.8)	
Behavior, *n* (%)					0.41
B1(nonstricturing, nonpenetrating)	54 (28.4)	430 (27.8)	243 (24.1)	152 (28.4)	
B2 (stricturing)	68 (35.8)	554 (40.8)	412 (40.2)	216 (43.2)	
B3 (penetrating)	23 (12.1)	175 (11.3)	118 (11.7)	77 (14.4)	
B2 + B3	45 (23.7)	389 (25.1)	237 (23.5)	93 (17.3)	
Disease location at IUS image, *n* (%)	/				0.81
Ileocecal region		338 (21.8)	228 (22.6)	99 (18.4)	
Colon		710 (45.8)	462 (45.7)	254 (47.2)	
Small intestine		500 (32.3)	320 (31.7)	185 (34.4)	
Simplified CDAI (mean ± SD)	7.1 ± 4.6	7.0 ± 4.6	6.8 ± 4.3	7.4 ± 5.0	0.13
hsCRP, mg/L (mean ± SD)	21.6 ± 29.1	21.5 ± 28.8	26.0 ± 31.7	17.6 ± 20.3	0.36
SES-CD, median (IQR)	6 (2.8–7.9)	6 (0.7–18.9)	6 (1.7–8.6)	6 (1.5–10.8)	0.87
Smoking habit, *n* (%)	41 (21.3)	338 (21.8)	251 (24.9)	87 (16.2)	0.03
Previous bowel surgery, *n* (%)	40 (20.8)	263 (17.0)	192 (19.0)	71 (13.2)	0.90
Medications, *n* (%)					
Corticosteroids at baseline	83 (43.2)	729 (47.1)	565 (55.9)	164 (30.4)	0.73
Mesalamine	32 (16.7)	237 (15.3)	190 (18.8)	47 (8.7)	0.01
Thiopurines	77 (40.1)	601 (38.8)	509 (50.4)	92 (17.1)	0.83
Biologics	104 (54.2)	906 (58.5)	467 (46.2)	439 (81.5)	0.06

*IUS* intestinal ultrasound, *SD* standard deviation, *CD* Crohn’s disease, *CDAI* Crohn’s disease activity index, *hsCRP* hypersensitive C-reactive protein

#### Deep learning architecture

**Fig. 2:**
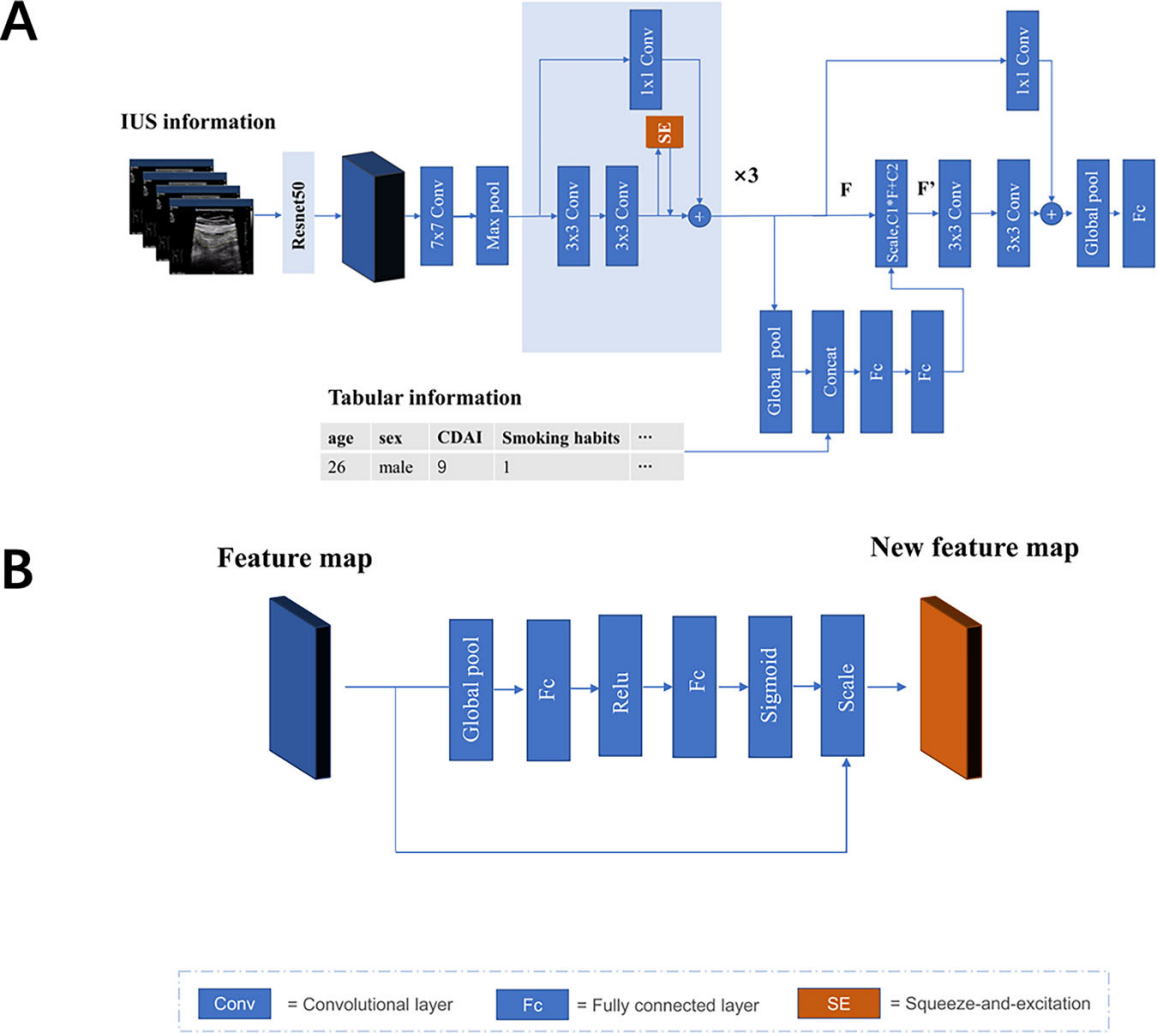
Scheme of a CNN. **A** A flowchart of the CNN model workflow. The model consists of three parts: the IUS image feature extraction part with an SE module, the IUS image and tabular data fusion part, and the classifier part. **B** A SE structure diagram. Input is the rough feature map collected from the previous step, and output is the new feature map scaled after the Sigmoid activation function, which calculates the weight coefficient of each channel. IUS, intestinal ultrasound; CDAI, Crohn’s disease activity index; FC, full connection; ReLU, rectified linear unit, one activation function

Image and tabular clinical data of patients were combined and used to classify mucosal healing and non-mucosal healing. A convolutional neural network (CNN) was constructed, with the main model structure illustrated in Fig. 2. A pre-trained ResNet50 model was used to extract features from IUS image data [18]. Then, the feature map was processed using three ResNet residual blocks. Squeeze-and-excitation (SE) was applied in the residual block to scale image features, directing the deep learning model to channel features [19]. Finally, image and tabular information were fused in three steps: first, features of images and tabular variables were combined; second, the dynamic affine feature map transform structure dynamically scaled and offset the feature graph in the convolution module [20]; finally, the full connection layer was used to classify mucosal healing and no mucosal healing.

Furthermore, a 5-fold cross-validation within the training cohort was performed, aiming to avoid model overfitting and improve reliability. To address the influence of complex intestinal fistula data on the model’s efficacy, we extracted a subset of the complex intestinal fistula images (56 images) for fine-tuning the model. During the fine-tuning process, we exclusively used this subset of data for training the model to adapt to the more complex situation. Repeatedly, we tested the performance of the fine-tuned model with 5-fold cross-validation. Then we evaluated the model’s performance on the separated test cohort.

The predictive performance was also assessed at the patient level. According to the rule applied in our study, if all IUS images of a patient were predicted as achieving mucosal healing, then that patient was classified as achieving mucosal healing. Conversely, if any of the IUS images of a patient were predicted as not achieving mucosal healing, then the patient was classified as not achieving mucosal healing. We compared these predictions made by our model with the ground truth determined from endoscopic assessment.

### Gradient-weighted class activation mapping (Grad-CAM) visualization

While deep neural networks can achieve good performance, they are often challenging to interpret due to their complexity. To address this, we used Grad-CAM to explain our prediction model because it generates class-discriminative localization maps without requiring any architectural modifications, making it well-suited for visualizing the key regions attended by our CNN-based model. This visualization enhances the interpretability of the model’s decision-making process and supports the reliability of the predictions in our task [21]. Although we fused image features and clinical information in the network structure, the fused features were weighted towards the image features as attention after the Dynamic affine feature map transform structure, with the model predominantly relying on the image features in the decision-making process.

### Statistical analysis

Descriptive statistics were reported as numbers (percentages) for categorical variables, mean ± SD for continuous normally distributed variables, and median (range) for continuous non-normally distributed data. Differences in clinical and laboratory data between the mucosal healing and no mucosal healing groups were calculated using the *t*-test, Mann–Whitney *U*-test, or chi-square test, as appropriate based on data distribution. The performance of the CNN model was described in terms of sensitivity, specificity, positive predictive value (PPV), negative predictive value (NPV), and accuracy. Receiver operating characteristic (ROC) curve analysis was performed, and the area under the curve (AUC) was calculated. MedCalc Software 19.5.3 (MedCalc Software Ltd) and Python 3.7 (Python Software Foundation) were used for statistical analysis. A *p* value < 0.05 was considered significant.

## Results

### Study population characteristics

A total of 238 patients met our inclusion criteria during the study period. Of these, 11 patients did not complete endoscopy examinations, 10 patients had incomplete IUS examinations, and 27 patients underwent surgeries during the follow-up period, resulting in a total of 48 patients being excluded. Accordingly, 190 patients were recruited. Tabular information, including demographic, clinical, laboratory, and medication characteristics, is shown in Table 1. The patient age was averaged as 32.3 ± 14.1 years, with an average disease duration of 73.4 ± 58.1 months. Forty-two (22.1%) patients had ileal disease, 43 (22.6%) had colon disease, and 105 (55.3%) had ileocolonic disease. For medications, 32 (16.7%) received mesalamine, 77 (40.1%) received thiopurines, and 104 (54.2%) received biological therapy. There were 83 (43.2%) patients who also received corticosteroids at baseline.

A total of 1548 baseline IUS images of diseased bowel segments were collected from the recruited patients. As per the image, there were 338 (21.8%) IUS images of the ileocecal region, 710 (45.8%) of the colon, and 500 (32.3%) of the small intestine. Additionally, 338 (21.8%) images were from patients who are current or ex-smokers. Medications intended to treat the disease included steroids at baseline (47.1%), mesalamine (15.3%), thiopurines (38.8%), and biologics (58.5%), with some cases involving combination treatments.

Based on the evaluations at 12 months after treatment initiation, 97 (51.1%) patients achieved mucosal healing while 93 patients (48.9%) did not achieve mucosal healing, resulting in 1038 IUS images of mucosal healing and 510 images of no mucosal healing. Patients and images were sequentially separated into training (120 patients, 1010 images) and test (70 patients, 538 images) cohort (Fig. 1). There was no significant difference in the percentages of mucosal healing images in training and test cohorts (692/1010 (68.5%) in the training cohort vs. 346/538 (64.3%) in the test cohort, *p* = 0.1).

### Predictive performance of the CNN model

**Fig. 3:**
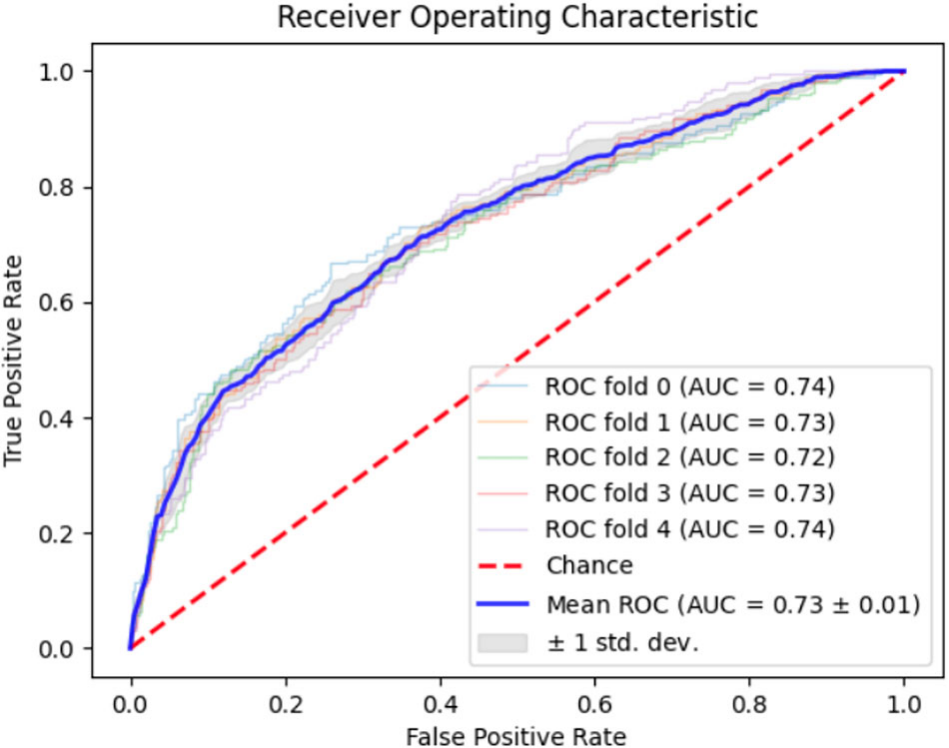
ROC curve of the CNN model

Using only IUS images to develop the CNN model for segmental mucosal healing prediction, the mean AUC of the 5-fold internal cross-validation is 0.66 (95% CI: 0.61–0.73) (Supplementary Table 1). Then, we incorporated clinical and laboratory tabular data along with the IUS images (Fig. 2). In the training cohort, the mean AUC of the 5-fold internal cross-validation was 0.77 (95% CI: 0.71–0.83). In the test cohort, the AUCs of the five deep learning models ranged from 0.72 to 0.74, with a mean value of 0.73 (95% CI: 0.68–0.78) (Fig. 3). The mean accuracy, sensitivity, specificity, PPV, and NPV for predicting segmental mucosal healing were 68.6% (95% CI: 66.8–70.3%), 68.1% (95% CI: 65.8–70.3%), 69.5% (95% CI: 66.5–72.4%), 80.0% (95% CI: 78.4–81.6%), and 54.8% (95% CI: 52.8–56.8%), respectively (Table 2). For comparision, the AUC of baseline IBUS-SAS to predict segmental mucoal healing is 0.67 (95% CI: 0.69–0.76) with a cutoff value of 34.5, and the accuracy, sensitivity, specificity, PPV, NPV are 62.8% (95% CI: 54.5–68.1%), 72.6% (95% CI: 64.5–77.8%), 57.8% (95% CI: 53.5–61.7%), 46.9% (95% CI: 41.7–52.3%), and 80.5% (95% CI: 73.3–83.8%) (Table 2).

**Table 2:** Performance of the deep-learning model and IBUS-SAS score for predicting segmental mucosal healing on 5-fold validation in the test cohort

		AUC	Accuracy (%)	Sensitivity (%)	Specificity (%)	PPV (%)	NPV (%)
Deep-learning model	Fold 1	0.74	72.0	72.7	70.8	81.8	59.1
	Fold 2	0.73	67.7	67.5	68.2	79.2	53.9
	Fold 3	0.72	67.4	65.8	70.3	79.9	53.3
	Fold 4	0.73	69.0	68.1	70.8	80.8	55.3
	Fold 5	0.74	66.4	66.1	67.2	78.3	52.4
	Mean	0.73	68.9	68.1	69.5	80.0	54.8
IBUS-SAS	/	0.67	62.8	72.6	57.8	46.9	80.5

*AUC* area under the receiver operating characteristic, *PPV* positive predictive value, *NPV* negative predictive value, *IBUS-SAS* international bowel ultrasound segmental activity score

On the patient level, the predictive value of the CNN model was calculated by aggregating the judgment of individual IUS images from the same patient. The mean sensitivity and specificity of predicting mucosal healing were 37.7% (95% CI: 23.5–53.6%) and 88.8% (95% CI: 73.4–96.9%), respectively.

### Visualizing the deep learning process by Grad-CAM

**Fig. 4:**
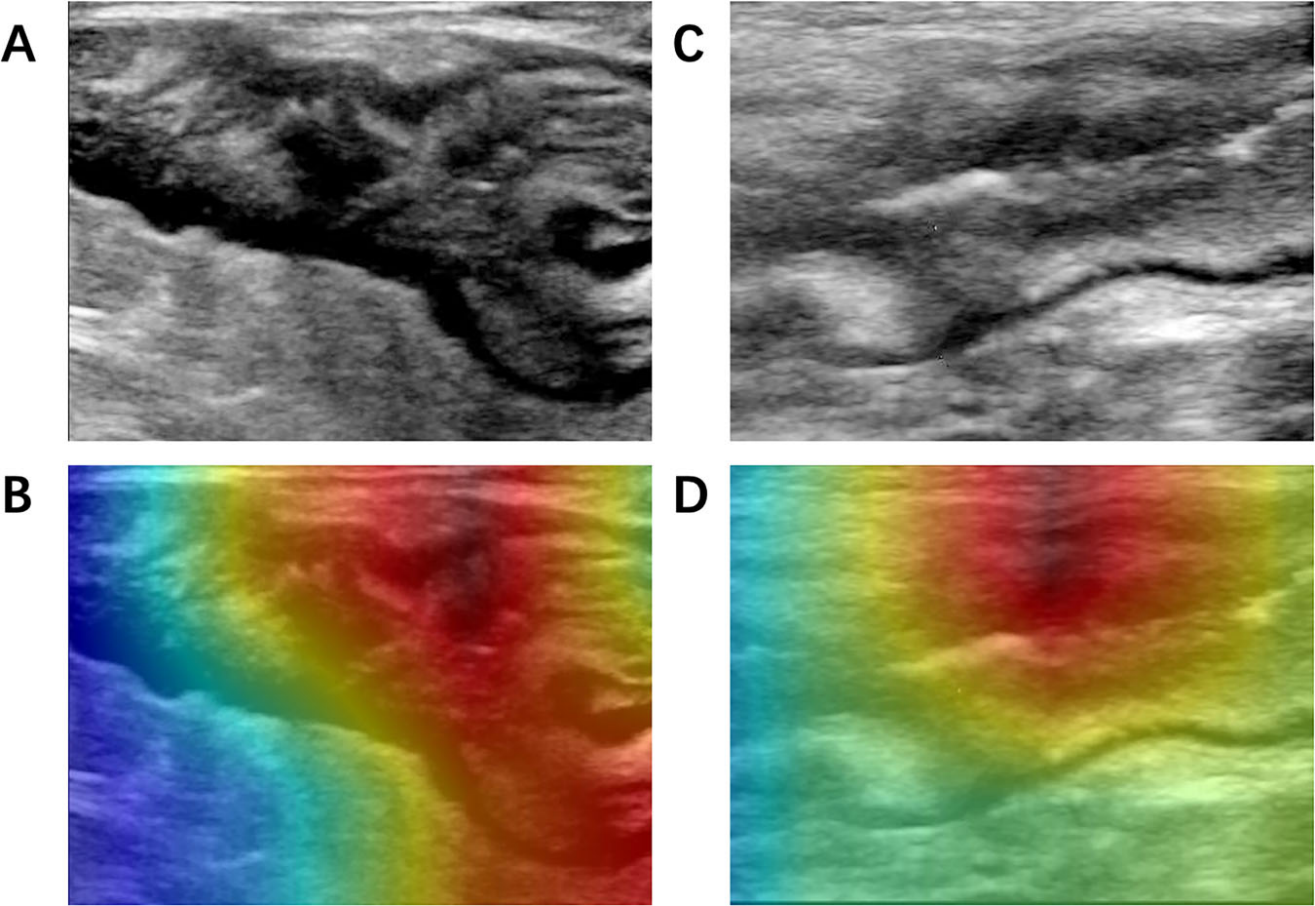
Applying Grad-CAM to improve interpretability. **A**, **C** IUS images; **B**, **D** the corresponding heat maps calculated by the Grad-CAM method, with warmer colors indicating regions activated by the CNN model with larger predictive significance, and colder colors indicating regions with less predictive significance. Grad-CAM, gradient-weighted class activation mapping

To determine whether the CNN model focused on features related to the state of the intestinal wall, we used the Grad-CAM method [21] to track the information extracted by the model. Figure 4 shows that our classifier can accurately capture relevant features of the most diseased intestinal wall, the serous surface, and the surrounding mesentery.

## Discussion

In the current study, we developed a deep-learning algorithm capable of predicting mucosal healing primarily based on ultrasound images and clinical information in CD patients, achieving an AUC of 0.73 and a PPV of 80.0%, indicating its potential utility in clinical practice. To our knowledge, this is the first ultrasound-based AI model to predict treatment response in CD.

Identifying patients most likely to respond well to treatment is critical for determining an effective treatment regimen. Numerous studies have aimed to identify potential clinical and laboratory predictors of poor treatment responsiveness; however, the practical implications for daily practice remain limited [5, 6, 22–27]. For example, from a clinical perspective, factors such as long disease duration, young-onset age, perianal disease, colon involvement, structuring phenotype, and smoking habit have been reported, with controversy regarding be association with a reduced probability of treatment success [22]. Several studies have also examined the use of laboratory tests, such as CRP and perinuclear antineutrophil cytoplasmic antibodies, as predictors of treatment effectiveness, also yielding inconsistent results [5, 6, 28]. Recently, some preliminary studies assessing apoptotic gene polymorphisms, circulating cytokines, miRNAs, and gene expression levels as predictors of thiopurine and biologic treatment efficacy have shown promise [23, 24, 26, 27]. However, these results need validation in larger cohorts.

The use of pretreatment imaging methods to predict treatment response in CD is less frequently reported. Rimola et al analyzed features of pretreatment MRI images of 58 patients to predict responsiveness to anti-TNF treatment, demonstrating that location and creeping fat were negative predictors of good treatment response [29]. A prediction model based on MRI showed a concordance rate of 0.67 to treatment failure at the internal validation [30]. Atreya et al used confocal laser endomicroscopy to count mTNF-positive cells, which are associated with treatment responsiveness [31]. Several recent studies have shown the predictive value of IUS. Chen et al used BWT and SWE values to predict the treatment response of anti-TNF therapy in 30 CD patients, achieving an AUC of 0.683 [4]. Albshesh et al showed that terminal ileum transmural thickness is associated with infliximab therapy success in 60 CD patients with terminal ileum involvement, with an AUC of 0.844 [8]. Dolinger et al demonstrated the association of pretreatment small bowel ultrasound findings and clinical and biomarker response at week 14 of infliximab treatment in 13 CD patients [9]. A recent study used baseline IBUS-SAS to predict mucosal healing and achieved an AUC of 0.70, which is consistent with our result [32]. Conclusively, most prior studies utilized small sample sizes or lacked external validations, resulting in insufficient and inconclusive evidence to support the clinical application of their findings [22].

Our results contribute to the growing evidence demonstrating the potential power of the deep learning algorithm for outcome prediction in CD. Earlier research above has indicated that intestinal images contain valuable information for predicting treatment responsiveness, which may be captured by deep learning neural networks in ways not detectable by human eyes. While artificial intelligence has made great breakthroughs in fields such as breast, neural, and chest imaging, applications in inflammatory bowel disease are still emerging, with most studies focusing on endoscopic techniques like regular or capsule endoscopy [33, 34]. The complexity of CD requires comprehensive, integrated information for clinical decision-making, making it an ideal candidate for artificial intelligence applications. For example, Con et al demonstrated that deep learning models, based on clinical and laboratory data, outperform conventional algorithms in predicting clinical remission, achieving an AUC of 0.754 in 146 patients. [35]. Similarly, a recent study applied deep learning analysis based on capsule endoscopy videos to predict the need for biological therapy in 101 patients, receiving an AUC of 0.86 [36]. In contrast, our study leverages two-dimensional imaging data to provide detailed predictions for individual bowel sections. By using mucosal healing as the ground truth, our model offers a more objective and widely accepted endpoint compared to “soft” clinical markers, enhancing its practical utility.

Despite the impressive capabilities of deep learning models, predicting clinical outcomes remains challenging and complex. As such, it is understandable that our model’s performance may not match the high accuracy of some diagnostic models. Nevertheless, the high PPV of our model underscores its effectiveness in identifying mucosal healing, which is highly valuable in clinical practice. The use of Grad-CAM visualization further validates the reliability of our model by highlighting its focus on key features of diseased intestinal sections.

To address overfitting—a common issue in deep learning where a model performs well on training data but poorly on new data—we employed several strategies. We implemented five-fold cross-validation, which partitions the dataset into five parts, using four parts for training and one for testing in each iteration. This approach mitigates evaluation errors from data partitioning randomness and reduces the risk of overfitting. Additionally, we utilized data augmentation techniques, such as random rotation and flips, to enhance data diversity and complexity, thereby helping the model learn more features and patterns.

Our study has some limitations. As a retrospective analysis, the IUS examinations were conducted by a single radiologist who was not blinded to the clinical and laboratory data, potentially introducing operator dependency. Moreover, excluding patients who underwent surgeries or interventions before the 12-month assessment may introduce selection bias. Finally, the model was developed using IUS data from a single medical center, necessitating further validation with larger datasets from other institutions.

In summary, we developed a deep learning model based on IUS images to predict mucosal healing in CD with notable accuracy. This model has the potential to identify patients who may respond poorly to treatment, thereby supporting timely clinical decision-making and promoting suitable personalized treatment strategies for gastroenterologists. Further validation and improvement of this model with more multi-center, real-world data are needed.

## Supplementary information

ELECTRONIC SUPPLEMENTARY MATERIAL

## Data Availability

The data that support the findings of this study are available on request from the corresponding author, Q.Z., upon reasonable request.

## Abbreviations

AUC: Area under the curve
BWT: Bowel wall thickness
CD: Crohn’s disease
CNN: Convolutional neural network
CRP: C-reactive protein
ECCO-ESGAR: European Crohn’s and Colitis Organisation and the European Society of Gastrointestinal and Abdominal Radiology
Grad-CAM: Gradient-weighted class activation mapping
IUS: Intestinal ultrasound
NPV: Negative predictive value
PPV: Positive predictive value
ROC: Receiver operating characteristic
SE: Squeeze-and-excitation
SES-CD: Simplified Endoscopic Score for Crohn’s Disease
