# Supplementary material for: Predicting mucosal healing in Crohn’s disease: development of a deep-learning model based on intestinal ultrasound images
Source: Insights Imaging. 2025 Jun 16;16:125. doi: 10.1186/s13244-025-02014-5 (PMC12170472; doi:10.1186/s13244-025-02014-5)
Supplement: Supplementary file 1 — ELECTRONIC SUPPLEMENTARY MATERIAL [file 13244_2025_2014_MOESM1_ESM.pdf]

# Predicting mucosal healing in Crohn's disease: Development of a deep-learning model based on intestinal ultrasound images

# ELECTRONIC SUPPLEMENTARY MATERIAL

**Supplementary Table1 Five-fold cross-validation result of the deep learning model based on intestinal ultrasound image alone**

| | AUC | Accuracy (%) | Sensitivity (%) | Specificity (%) | PPV (%) | NPV (%) |
|---|---|---|---|---|---|---|
| Fold 1 | 0.68 | 63.6 | 61.7 | 67.3 | 78.6 | 47.4 |
| Fold 2 | 0.69 | 59.7 | 57.8 | 65.9 | 84.8 | 32.1 |
| Fold 3 | 0.61 | 54.3 | 51.8 | 61.2 | 79.1 | 30.9 |
| Fold 4 | 0.67 | 62.5 | 62.7 | 62.3 | 72.2 | 51.6 |
| Fold 5 | 0.66 | 63.1 | 60.8 | 68.8 | 83.0 | 41.3 |
| Mean | 0.66 | 60.6 | 58.9 | 65.1 | 79.5 | 40.7 |

Abbreviations: AUC, area under the receiver operating characteristic; PPV, positive predictive value; NPV, negative predictive value.
